# Susceptibility to Smoking among Adolescents and Its Implications for Mexico’s Tobacco Control Programs. Analysis of the Global Youth Tobacco Survey 2003–2004 and 2006–2007

**DOI:** 10.3390/ijerph6031254

**Published:** 2009-03-23

**Authors:** Raydel Valdés-Salgado, Luz Myriam Reynales-Shiguematsu, Eduardo C Lazcano-Ponce, Mauricio Hernández-Avila

**Affiliations:** 1 Instituto Nacional de Salud Pública Avenida Universidad 655, Santa Maria Ahuacatitlan, Cuernavaca 62508, Morelos, Mexico; E-Mails: lreynales@insp.mx (L.M.R.); elazcano@insp.mx (E.C.L.); 2 Institute for Global Tobacco Control, Johns Hopkins Bloomberg School of Public Health. 615 N Wolfe Street, Room W6501, Baltimore, MD 21205, USA; 3 Ministry of Health. Lieja No. 7 Col. Juárez Deleg, Cuauhtémoc, Mexico D.F. 06600, Mexico; E-Mail: mhernan@correo.insp.mx (M.H.)

**Keywords:** Susceptibility to smoking, adolescents, GYTS, tobacco control and prevention, Mexico

## Abstract

Smoking prevention efforts should either prevent target groups from becoming susceptible to smoking or prevent susceptible adolescents from progressing to becoming regular smokers. To describe the prevalence of susceptibility to smoking among never smoker students from cities that applied the GYTS in 2003 and 2006. The GYTS uses a two-stage cluster sample survey design that produces representative samples of students aged 12–15 years enrolled in public, private, and technical schools. The survey was undertaken at 399 schools in 9 cities. The GYTS surveyed 33,297 students during the academic years 2003–04 and 2006–07. Among never smokers, about 25% are likely to initiate smoking in the next 12 months. There are no differences in susceptibility to smoking by gender. When comparing results from 2003 and 2006, the susceptibility index has not changed, but for one city. The GYTS results are useful for monitoring susceptibility to smoking among adolescents and provide evidence for strengthening the efforts of tobacco control programs in Mexico.

## 1. Introduction

Today it is well-accepted within the global health community that active smoking causes more than 25 specific deadly diseases [1] and that the tobacco epidemic is moving towards the developing world [2,3]. Most of the burden of disease attributable to smoking occurs among adults [4], however the problem originates in the teenage years when most smokers have their first experience with cigarettes. In developed nations, the vast majority of smokers begin using tobacco products well before the age 18 [5] and worldwide nearly one-quarter of young people who smoke had their first cigarette before the age of ten [6].

The World Health Organization Framework Convention on Tobacco Control (FCTC) [7] and the MPOWER report [8] recommend concrete actions to reduce tobacco use which are the promotion of smoke-free environments, advertising bans, restrictions on the accessibility of cigarettes to minors, cessation assistance, education campaigns, and increased cigarette prices through excise taxes. These measures impact the tobacco epidemic in at least three ways: among adults, by reducing the number of current smokers and preventing ex-smokers from relapse; and among young people, mainly – but not exclusively – by hindering initiation. When addressing youths, our efforts should either prevent target groups of non-smokers from becoming susceptible to smoking or prevent susceptible adolescents from progressing to regular smokers.

Since at least the early 1970s, many authors have suggested that smoking uptake behavior in adolescence is a multi-step developmental process that can be broadly described as a continuum from never smokers to experimenters to established smokers; the latter group including both occasional and daily smokers. Despite the differences in definitions and measurements, and to some extent overlap in stage conceptualizations and blurred boundaries of the stages, agreement exists on the basic premise of the stages. A theoretical discussion on the development of adolescent smoking is beyond the scope of this paper and we recommend other insightful reviews on the issue that have been published elsewhere [9–11].

In this paper, the main focus is on the concept of susceptibility to smoking [12]. Susceptible adolescents are those who are currently not smoking, but who are cognitively predisposed or motivated to start smoking in the future. A measure of susceptibility to smoking includes the participant’s intentions and expectations for future behavior. Thus, susceptibility to smoking is defined as the absence of a firm decision not to smoke. Likewise, non-susceptibility is defined as the existence of a determined decision not to smoke [13].

The predictive validity of susceptibility to smoking was tested in a longitudinal design with a nationally representative sample of adolescents in the United States. In that study, the baseline measure of susceptibility was a stronger predictor of experimentation than was the presence of smokers among family and best friends [14]. However, results from another longitudinal study suggest that susceptibility to smoking is not an independent risk factor for ever smoking but rather a potential mediating variable [15].

In the context of Mexico, susceptibility to smoking among adolescents has been previously reported in publications derived from the application of the Global Youth Tobacco Survey (GYTS) [16–18]. More recently, another study described susceptibility to smoking and associated factors among students attending public schools in 10 Mexican cities [19].

Currently, the smoking behavior of young people in Mexico is at the center of attention of both the tobacco industry and the tobacco control advocates. Because of its age structure, Mexico still has a young population. Data from the most recent Count of Population and Housing (2005) shows that 30.6% of Mexicans are 14 years old or younger. Particularly, there are 10.7 million people between the ages of 12 and 16 [20]. That segment of the population is a valuable target for the tobacco industry because it represents potential smokers in future decades. From a public health perspective, to prevent that group from smoking represents the success of current tobacco control efforts at national and local levels and the beginning of the end of the tobacco epidemic in the country by reducing the flow of new smokers.

The main objective of this paper is to describe susceptibility to smoking among never smoker students from cities that participated in two waves of the GTYS during the academic years 2003–2004 and 2006–2007.

## 2. Methods

The GYTS uses a two-stage cluster sample survey design that produces representative samples of Mexican students in grades associated with ages 12–15 years enrolled in private, public, and technical schools. The sampling frame included all schools containing any of the identified grades. In the first stage, the probability of a school being selected was proportional to the number of students that had enrolled in the specified grades. In the second sampling stage, classes within the selected schools were randomly selected. All students attending school the day the survey was administered in classes were eligible to participate.

For analysis, a weighting factor was applied to each student record to adjust for non-responses and variation in the probability of selection at the school, class and student levels. All tables present the estimate and its 95% confidence interval (95% CI). Differences in proportions were considered statistically significant at the *p*<0.05 level assessed by non-overlapping confidence intervals. In order to compare the results from the two applications in each city, we adjusted by age due to small but significant differences in the mean age of the samples of the first and the third application (In most of the cities the sample in 2003–2004 was slightly younger because that survey was conducted at the beginning of the academic year while the second application was conducted during the second half of the academic year). Age-adjusted prevalence odds ratios (OR) are presented when comparing the two applications of the survey. All analyses were performed with STATA software, version 10 [21].

The GYTS has been applied in Mexico three times: 2003–2004, 2004–2005, and 2006–2007. Since the first wave, the selection of participant cities was planned to be informative of tobacco use throughout the country, meaning that each application includes cities from the north, center, and southeast region. All waves of the GYTS have used a common sampling methodology and a core questionnaire, therefore we can compare across cities and between cities that have been surveyed twice.

Main definitions in this paper are: *smoker,* a student who smoked cigarettes on one or more days in the 30 days preceding the survey. *Never smoker*, students who had never smoked cigarettes, not even one or two puffs. Proportion of *never smokers susceptible to smoking* is defined as 100%, minus the proportion of never smokers who answered “definitely not” to the question: “If one of your best friends offered you a cigarette, would you smoke it?”, and answered “definitely not” to the question: “At any time in the next 12 months do you think you will smoke a cigarette?”

The GYTS is conducted in Mexico by the National Institute of Public Health (INSP) in collaboration with the National Council against Addictions and the State Council against Addictions in each participant city. The Pan American Health Organization (PAHO) and the Centers for Disease Control and Prevention (CDC) provided financial and technical assistance and the INSP also provided funds to partially support this endeavor.

## 3. Results

Table 1 shows characteristics of the sample. In total, 33,297 students of first, second, and third grade of secondary level from 399 private, public, and technical schools were surveyed. Approximately half of the respondents were females (51.5%). The nine cities analyzed here are those with two measurements, one in the first wave (2003–2004) and another in the third wave (2006–2007): Mexico City, Juarez, Nuevo Laredo, Tijuana, Cuernavaca, Guadalajara, Puebla, Chetumal, and Tapachula.

Table 2 shows overall smoking prevalence for each city that participated in both applications. In general, figures for smoking prevalence ranged from 11% to 28%. During the first wave, the lowest prevalence was observed in Tijuana 11.5% (9.3% to 13.7%) and the highest was in Puebla, 25.4% (20.0% to 30.8%). In the second application, again the lowest prevalence was observed in Tijuana 13.0% (11.2% to 15.2%) and the highest was in Puebla, 27.5% (24.9% to 30.2%). Only three cities showed significant reduction in smoking prevalence: Juarez: age-adjusted OR=0.88 (95% CI 0.80–0.97), Nuevo Laredo: age-adjusted OR=0.90 (95% CI 0.82–0.99) and Chetumal: age-adjusted OR=0.88 (95% CI 0.80–0.97).

In Table 3 we can see the prevalence of non-smokers who are susceptible to smoking, by gender. There are no differences in the proportion of “never smoker susceptible to smoke” between males and females. An unexpected finding was that in some states the proportion of non-smoker females that are susceptible to initiate smoking is higher than that among their masculine counterparts. Although the differences are not statistically significant, it is interesting to mention some examples: in the first application, Mexico City, Guadalajara, Cuernavaca, Juarez, Nuevo Laredo, Chetumal, and Puebla; in the second, Tijuana and, again, Puebla.

In Table 4 we compare the city specific overall estimates of susceptibility in each application. In general, one out of five non-smokers was susceptible to smoking within the next 12 months.

Only in Tapachula (2003–2004) the proportion of susceptible to smoking was 18.1% (16.0% to 21.6%). For the rest of the cities, that proportion is predominantly above 23%. The highest values were observed in Puebla 30.4% (26.7% to 34.1%), Guadalajara 28.1% (25.2% to 31.0%), and Cuernavaca 27.5% (23.8% to 31.2%) during the first application. Most recently, the highest proportions were observed in Puebla 31.0% (27.4% to 34.9%), Mexico City 29.4% (26.2% to 32.9%), and Cuernavaca 27.6% (24.0% to 31.5%). Table 4 also shows that there was almost no change in the prevalence of “never smokers susceptible to smoking” when comparing the two applications three years apart. Only for Chetumal did we find a significant 11% reduction in the likelihood of identifying non-smokers who are susceptible to smoking, age-adjusted OR=0.89 (95% CI 0.82–0.97) as compared to the first survey.

## 4. Discussion

Three major problems are unveiled in this paper with regard to susceptibility to smoking. First, approximately one fourth of never smokers are susceptible to start smoking. Second, there are no differences in susceptibility to smoking by gender. Third, we found no evidence of reduction in susceptibility to smoking in eight out of nine cities when comparing two applications of the GYTS.

When comparing results from two surveys three years apart, we observed that the prevalence of “never smokers susceptible to smoking” seemed to be firmly about 25% in most cities. Therefore, our findings in this paper are quite disturbing because they point in the direction of an increase in the short term in the actual number of adolescents who smoke.

The analysis of earlier applications of the GYTS in Mexico provides some clues about what features are associated with susceptibility to smoking. Data from 42,024 students surveyed during 2003–2005 showed that some of the factors that enhance susceptibility to smoke among adolescents are having positive thoughts toward smokers, possession of items marketed by the tobacco industry, exposure to environmental tobacco smoke, having a best friend who smokes cigarettes, and underestimation of health effects of tobacco use and nicotine addiction. On the other hand, factors that constrain the behavior are perceptions that she/he is too young to smoke, recognition that tobacco is a drug, and knowledge that cigarette smoke is harmful along with discussion within the family about smoking’s harmful effects [22].

Another large study conducted in secondary schools from several Mexican cities also reported prevalence of susceptibility between 25% and 30% and lack of difference by gender. In addition, they identified some psychological variables associated with susceptibility, namely, low self-esteem, seeking new sensations, and favorable attitude towards smoking. Particularly for women, they found an association between susceptibility and permissibility of smoking at home and tobacco consumption by parents [19]. The conclusions from this study, along with that of the GYTS, replicate findings previously reported in different contexts, particularly with regard to the relevance of parental and household influences [9,13] as well as the perceived smoking behavior of peers [23,24]. The presence of other smokers in the family as a contributing factor to susceptibility to smoking among Mexican adolescents seems reasonable because at least for Mexico City two studies have documented the exposure of minors to SHS in homes [25,26].

In the Mexican social context other factors might also encourage adolescents to smoke. For example, a study conducted in 2006 documented the closeness of secondary schools to billboards advertising tobacco products and that tobacco products, including single cigarettes, were available to minors in streets around the schools [27]. In spite of the fact that the sale of cigarettes to people under age 18 years old is illegal in Mexico, selling to minors is still a serious problem that undermines other tobacco control efforts. Traditionally, the problem has been so big that a 2002 study of convenience stores and other points of purchase in Mexico City observed that in 73% of them a participating minor in the study succeeded in buying cigarettes [28].

The GYTS also shows differences in smoking prevalence across cities and in-depth discussions of that issue have been published elsewhere [16–18,22,29]. Three out of the four largest Mexican cities are included in this comparison and although not statistically significant change in smoking prevalence was documented, it is alarming that figures for Mexico City and Puebla are higher in 2006 than in 2003, while in Guadalajara there seems to be a downward trend in tobacco use among secondary students. In this study we found that the lowest prevalences are observed in border cities. Tijuana and Nuevo Laredo in the northern region, and Chetumal and Tapachula in the southeast, showed in both applications prevalences below 20%. We also know from the application of the GYTS in 2005 that the smoking prevalence in other northern cities such as Monterrey (18.7%) and Culiacan (10.5%) are below 20% [17]. These findings are different from the pattern that has been described for the general population, where the highest rates of smoking prevalence are observed in north and central Mexico, while states in the south have historically showed the lowest rates of tobacco consumption [30].

Also, we found that only the border cities of Nuevo Laredo, Juarez, and Chetumal showed a significant reduction in smoking prevalence between 2003 and 2006. Therefore, we hypothesized that broader efforts to protect youth from initiation with illegal drugs are the main reason behind the remarkable reduction in smoking prevalence in these three cities. In last few decades some Mexican states - particularly those in border regions- have been seriously affected by the presence of drug cartels and other drug-related social problems. Because tobacco use in adolescence is associated with higher probabilities of initiation with illegal drugs [31], local authorities, the schools, and other community-based organizations might have identified anti-smoking efforts as a successful way to prevent drug use among young people. If so, these preventive local efforts deserve more attention and perhaps can be used as models to enhance other local tobacco control programs.

With respect to the lack of differences in susceptibility by gender, it is consistent with recent reports describing an alarming worldwide increase in smoking rates among women [32–36]. The traditional gender gap in smoking prevalence is closing among new generations of Mexicans and in this study we found no reason to think that adolescent girls are still more protected from smoking than adolescent boys. In previous reports we already showed that smoking prevalence in Mexican adolescents is similar among males and females [16–18] and here we did not find differences in susceptibility to smoking by gender in any of the surveyed cities.

Studies have shown that a person's risk for using tobacco products is closely associated with that person's exposure to tobacco marketing [33] and the tobacco industry aggressively markets younger females in high-population countries and probably Mexico isn’t an exception. Advertising, promotions, and sponsorship are some of the strategies to weaken cultural opposition to women using tobacco and to reach this segment of the market the tobacco industry designs products and brands that are marketed specifically to women and explicitly encourage them to smoke [8]. Lack of differences by gender as shown by the GYTS in Mexico does not differ from what has been described by the GYTS globally [36]. To cope with this challenge, other authors have proposed that tobacco control programs need to counter current tobacco advertising and marketing practices aimed at young women with alternative images related to independence and overall self-image as well as educate girls about the effect of tobacco use on reproductive health outcomes [36].

It is difficult to explain why in most of the cities the proportion of “never smokers susceptible to smoking” did not change between the two applications of the survey. Indeed, an anti-smoking campaign was implemented in schools throughout the country during that period [37]. Literature is abundant in examples of how youth smoking prevention and control efforts have had mixed results. Particularly, school based curricula alone have been generally ineffective in preventing adolescents from initiating tobacco use [11,38]. In the last few years, Mexican health authorities have been working closer with the Ministry of Education in an effort to strength anti-tobacco messages in text books as well as provide more information about tobacco-related health outcomes and addiction to nicotine. However, the impact of such measures has not been fully evaluated yet.

However, when comparing the two surveys we also found some positive results. For example, for three cities we observed a reduction in smoking prevalence. Particularly, the city of Chetumal also reduced the prevalence of “never smokers susceptible to smoking” in that period. These findings suggest that some local tobacco control programs are making progress. In Mexico, the dominant stream in tobacco control has been a top-down approach with the implementation of the strongest measures at federal level. Perhaps it is time to pay not only more attention, but to stimulate local initiatives tailored to local challenges. Nevertheless, for the success of this alternative approach the necessity of both federal and local resources is warranted because in Mexico, as in the rest of the developing world, scarcity of resources competes against tobacco control efforts [39].

National tobacco control efforts in the past few years have been remarkable and we hope to see a significant impact on smoking behavior among Mexican smokers in the near future. Early in 2007 a new tax to cigarettes was applied [40] and in 2008 a national law of protection from exposure to secondhand smoke was passed and implemented [41]. These two measures are a clear message from the highest levels of the Mexican government of its commitment to tobacco control efforts. Thus, as tobacco control advocates and scholars we are now pushing for a comprehensive evaluation of the impact of such measures.

Regarding taxation, research from high-income countries suggests that, on the whole, young people are more price-responsive than older people [42]. Besides that, only comprehensive advertising bans can reduce tobacco consumption, because a limited set of advertising bans will have little or no effect [43].

Smoke-free policies produce benefits for non-smokers by eliminating passive smoking and making it easier for smokers to reduce or stop smoking [44]. It has been also shown in a longitudinal study that home smoking bans may promote anti-smoking attitudes among youth and reduce progression to smoking experimentation among youths who live with non-smokers [45]. Most adolescents who smoke have a low intensity pattern of consumption, but few cessation services are geared to that segment of smokers. Besides that, a review found that most of adolescents are unfamiliar with the concept of smoking-cessation program, or with other tools of methods that support quit attempts. Also, among them are concerns about confidentiality and parental involvement [38].

Regarding counter-advertisement, it is well known that ads focusing on the marketing practices of the tobacco industry increase anti-industry attitudes and, in turn, reduce smoking among youth [46]. In a comparison of communities with regular enforcement of restrictions of minors access to cigarettes to communities without regular enforcement, authors concluded that adolescents who had restricted access to tobacco products were less likely to become regular smokers [47].

All of the measures mentioned above must be fully implemented in a coordinated way to take advantages of potential synergies across interventions [48]. In Mexico, tobacco control efforts are coordinated by the Ministry of Health, in cooperation with scientific and academic institutions. Only in recent years, civil society organizations have increased their participation and some of them are now well recognized as tobacco control advocates. Mexico has recently built some basic capacity for tobacco control and comprehensive tobacco control programs exist at a national level and at local levels with varying degrees. However, compliance and enforcement remain unsolved matters. Likewise, evaluation of tobacco control policies is still an incipient field that has to mature along with the necessary skills for tobacco control advocacy and social mobilization.

In the regional context, Mexico’s proportion of “never smokers susceptible to smoking” resembles those observed in the Southern Cone and the Andean region. As shown in Tables 3 and 4, most Mexican cities show figures clearly above 20% and in Latin America such numbers are observed in Chile (28.4%), Argentina and Uruguay (25.1%), Bolivia (24.9%), Peru (24.4%), and Colombia (22.9%). Other countries from South and Central America as well as the Caribbean islands show lower numbers for susceptibility to smoking [49].

Findings in our study are subject to some limitations. This data applies only to youth aged 11–16 years old who attended school and were in the school on the day of survey administration. Additionally, the data is based on self reports possibly leading to under- or over-reporting behavior. On the other hand, use of common methodology, randomly selected samples, and weighted estimations made the figures presented here valid.

The GYTS, as part of the Global Tobacco Surveillance System [50], is a very useful tool to monitor tobacco use and to generate tobacco use related information, particularly in developing nations where otherwise updated and reliable information is scarce. In Mexico, the GYTS also works as state-level surveillance. Publication of results like those described in this paper are valuable not only for assessing tobacco use, but for designing more effective interventions at national and local levels to curb the current rates of tobacco use and susceptibility to smoking among adolescents.

## Figures and Tables

**Table 1.** Study population. GYTS Mexico 2003–2004 and 2006–2007.

Academic Year	City	Schools sampled	Students surveyed	% Females

2003–04	Mexico City	25	2,099	49.7
2003–04	Juarez	25	2,210	50.4
2003–04	Nuevo Laredo	21	1,416	51.2
2003–04	Tijuana	25	2,000	50.9
2003–04	Cuernavaca	25	2,075	54.8
2003–04	Guadalajara	24	2,059	53.5
2003–04	Puebla	23	1,888	49.8
2003–04	Chetumal	9	1,415	52.9
2003–04	Tapachula	24	2,155	50.0
2006–07	Mexico City	24	1,826	50.2
2006–07	Juarez	22	1,546	51.0
2006–07	Nuevo Laredo	24	1,414	51.2
2006–07	Tijuana	23	1,785	51.3
2006–07	Cuernavaca	23	2,001	54.7
2006–07	Guadalajara	24	2,093	53.5
2006–07	Puebla	22	1,767	49.7
2006–07	Chetumal	11	1,456	53.1
2006–07	Tapachula	25	2,092	50.1
	Total	399	33,297	51.5

**Table 2.** Smoking prevalence among Mexican adolescents. GYTS 2003–2004 and 2006–2007.

City	Prevalence of smoking during past 30 days (95% CI)	
	2003–2004	2006–2007

Mexico City	20.2 (16.3 – 24.1)	27.8 (24.0 – 31.9)
Juarez	22.6 (18.7 – 26.5)	17.7 (15.8 – 19.9)
Nuevo Laredo	16.5 (13.3 – 19.7)	14.2 (11.9 – 17.0)
Tijuana	11.5 (9.3 – 13.7)	13.0 (11.2 – 15.2)
Cuernavaca	20.8 (18.1 – 23.5)	21.7 (18.9 – 24.8)
Guadalajara	19.9 (15.2 – 24.6)	17.3 (14.8 – 20.2)
Puebla	25.4 (20.0 – 30.8)	27.5 (24.9 – 30.2)
Chetumal	17.9 (14.9 – 20.9)	14.6 (12.1 – 17.6)
Tapachula	13.8 (11.2 – 16.4)	16.3 (14.3 – 18.6)

**Table 3.** Susceptibility to smoking among never smokers, by gender. GYTS Mexico 2003–04 and 2006–07.

Academic Year	City	Males % susceptible to smoke (95% CI)	Females % susceptible to smoke (95% CI)
2003–04	Mexico City	22.0 (18.8 – 25.2)	27.5 (22.9 – 32.1)
2003–04	Juarez	21.5 (18.1 – 24.9)	23.8 (18.9 – 28.7)
2003–04	Nuevo Laredo	20.6 (17.7 – 23.5)	26.6 (21.1 – 32.1)
2003–04	Tijuana	22.8 (19.2 – 26.4)	21.3 (18.5 – 24.1)
2003–04	Cuernavaca	27.0 (22.2 – 31.8)	27.7 (22.2 – 33.2)
2003–04	Guadalajara	25.4 (21.8 – 29.0)	30.2 (25.6 – 34.8)
2003–04	Puebla	27.7 (23.6 – 31.8)	31.1 (25.2 – 37.0)
2003–04	Chetumal	24.5 (19.5 – 29.5)	27.1 (21.6 – 32.6)
2003–04	Tapachula	20.4 (16.0 – 24.8)	17.5 (14.3 – 20.7)
2006–07	Mexico City	29.0 (24.6 – 33.9)	27.8 (22.9 – 33.2)
2006–07	Juarez	23.2 (19.2 – 27.6)	24.0 (20.9 – 27.5)
2006–07	Nuevo Laredo	27.1 (22.2 – 32.7)	23.7 (18.7 – 29.7)
2006–07	Tijuana	18.8 (14.9 – 23.4)	22.1 (17.1 – 28.0)
2006–07	Cuernavaca	28.6 (22.6 – 35.5)	25.7 (20.3 – 32.0)
2006–07	Guadalajara	25.1 (19.9 – 31.1)	24.0 (20.9 – 27.3)
2006–07	Puebla	28.7 (24.9 – 32.8)	32.3 (26.8 – 38.4)
2006–07	Chetumal	22.3 (18.5 – 26.5)	20.4 (16.2 – 25.3)
2006–07	Tapachula	21.5 (16.4 – 27.6)	19.3 (15.6 – 23.7)

**Table 4.** Susceptibility to smoking among never smokers. GYTS Mexico 2003–2004 and 2006–2007.

City	Proportion of non smokers susceptible to smoke (95% CI)	
	2003–2004	2006–2007

Mexico City	25.1 (22.1 – 28.1)	29.4 (26.2 – 32.9)
Juarez	22.8 (20.1 – 25.5)	24.3 (21.3 – 27.6)
Nuevo Laredo	24.2 (20.8 – 27.6)	25.3 (21.8 – 29.1)
Tijuana	22.2 (20.2 – 24.2)	20.4 (16.9 – 24.5)
Cuernavaca	27.5 (23.8 – 31.2)	27.6 (24.0 – 31.5)
Guadalajara	28.1 (25.2 – 31.0)	24.5 (21.8 – 27.4)
Puebla	30.4 (26.7 – 34.1)	31.0 (27.4 – 34.9)
Chetumal	26.2 (22.5 – 29.9)	21.9 (19.0 – 25.1)
Tapachula	18.8 (16.0 – 21.6)	20.3 (17.3 – 23.6)
